# 645. Implementing a Structured Debriefing Tool to Improve Communication, Education, and Quality Initiatives

**DOI:** 10.1093/jbcr/irag033.482

**Published:** 2026-04-06

**Authors:** Krystle Ferreira, Kristy Gauthier

**Affiliations:** University of Utah Health Burn Center, Salt Lake City, UT; University of Utah College of Nursing, Salt Lake City, UT

## Abstract

**Introduction:**

Effective communication and team learning are critical to patient safety, yet post-event debriefing is often inconsistent or informal. This quality improvement project aimed to implement and evaluate a structured debriefing tool to enhance interdisciplinary communication, support ongoing education, and identify quality improvement opportunities in a Burn Trauma ICU.

**Methods:**

A multidisciplinary team developed a standardized tool based on TeamSTEPPS and AHRQ communication frameworks. The tool was piloted over two years in an ABA-verified Burn Center following high-acuity events. Staff received training on tool use, and data were collected via post-debrief surveys (confidence and education) and a dedicated debrief tool survey (perceptions of satisfaction, communication, and future use). Open-ended responses were analyzed thematically.

**Results:**

Debriefs were conducted after team dynamics/communication issues (n = 9), code blue/respiratory emergencies (n = 9), complicated admissions (n = 2), withdrawal of care (n = 1), and deep sedation/naloxone hydrochloride events (n = 1). In post-debrief surveys (n = 27), 55.5% of staff agreed or strongly agreed that prior education improved confidence in patient care, while 44.4% were neutral. In the debrief tool survey (n = 18), 89% reported satisfaction with the tool, 83% with the process, 89% felt heard, and 94% planned to continue using the tool. Although fewer responded to the communication-specific item, qualitative data reflected perceived improvements in communication and cohesion. Themes included: value of structured reflection and in-the-moment learning; improved cohesion and psychological safety; integration with safety briefings, time-outs, and checklists; desire for more solution-focused debriefs; pediatric-specific education needs (airway, equipment, medication safety); challenges during staff turnover; and recommendations for loop closure emails, Planning and PAUSE tools, and enhanced resilience support.

**Conclusions:**

Implementing a structured debrief tool improved communication, fostered informal education, and identified system-level improvements in a high-acuity burn setting. Integration of structured debriefing into clinical workflows supports a culture of reflection, collaboration, and continuous learning. Based on this project, we created an RN-led Quality Review Committee that identifies areas of need for quality improvement projects, education and training, and systematic changes, further strengthening unit safety and performance.

**Applicability of Research to Practice:**

This project demonstrates that nurse-led QI initiatives, supported by structured review processes, can generate measurable improvements in confidence, teamwork, and systems learning. Findings highlight opportunities for future research on debriefing’s impact on patient safety, staff resilience, and long-term performance outcomes.

**Funding for the study:**

N/A.

